# Something Old and Something New: Wedding Recombinant Inbred Lines with Traditional Line Cross Analysis Increases Power to Describe Gene Interactions

**DOI:** 10.1371/journal.pone.0010200

**Published:** 2010-04-16

**Authors:** Tarek W. Elnaccash, Stephen J. Tonsor

**Affiliations:** Department of Biological Sciences, University of Pittsburgh, Pittsburgh, Pennsylvania, United States of America; Leiden University Medical Center, Netherlands

## Abstract

In this paper we present a novel approach to quantifying genetic architecture that combines recombinant inbred lines (RIL) with line cross analysis (LCA). LCA is a method of quantifying directional genetic effects (i.e. summed effects of all loci) that differentiate two parental lines. Directional genetic effects are thought to be critical components of genetic architecture for the long term response to selection and as a cause of inbreeding depression. LCA typically begins with two inbred parental lines that are crossed to produce several generations such as F1, F2, and backcrosses to each parent. When a RIL population (founded from the same P1 and P2 as was used to found the line cross population) is added to the LCA, the sampling variance of several nonadditive genetic effect estimates is greatly reduced. Specifically, estimates of directional dominance, additive x additive, and dominance x dominance epistatic effects are reduced by 92%, 94%, and 56% respectively. The RIL population can be simultaneously used for QTL identification, thus uncovering the effects of specific loci or genomic regions as elements of genetic architecture. LCA and QTL mapping with RIL provide two qualitatively different measures of genetic architecture with the potential to overcome weaknesses of each approach alone. This approach provides cross-validation of the estimates of additive and additive x additive effects, much smaller confidence intervals on dominance, additive x additive and dominance x dominance estimates, qualitatively different measures of genetic architecture, and the potential when used together to balance the weaknesses of LCA or RIL QTL analyses when used alone.

## Introduction

Understanding the genetic basis of complex phenotypes, i.e. genetic architecture, is of fundamental importance both for modeling evolutionary change and for genetic manipulation of crop plants. Genetic architecture is a broad term for all factors that influence the determination of phenotype from genotype. It includes all genetic effects on traits: the number of genes, allelic effects, epistasis, pleiotropy, and genotype x environment interactions [1]. Knowledge of genetic architecture can inform us about the propensity to evolve (i.e. ‘variability’ *sensu* [2]) on all timescales.

Studies of genetic architecture have revealed that epistasis, i.e. interactions between loci, is a common component of most quantitative traits. For example, biomedical studies have shown an epistatic genetic basis for many human diseases [3] including diabetes [4], [5], Alzheimer's disease [6], obesity [7], cardiovascular disease [8] and schizophrenia [9]. Knowledge of the genetic basis of these diseases is important because epistatic traits can evolve in a fundamentally different way than additive traits [10]–[13]. Knowledge of gene interactions and genetic architecture is also important for building and evaluating models of evolutionary processes. All models of adaptation, population divergence and speciation assume a particular genetic architecture, but the assumptions vary wildly among models. At two ends of a spectrum, selection analyses used commonly in evolutionary ecology studies implicitly assume an additive genetic architecture [14], [15], while most studies of speciation assume an epistatic genetic architecture [16]–[18]. Does trait architecture change from additive to epistatic over some range of genetic distances or geographic distances? While patterns of the genetic architecture of inter-specific differentiation are becoming clear (e.g. Haldane's rule, Dobzhansky-Muller incompatibilities [19]–[21]), the genetic basis of differences within and among populations is more poorly understood and the genetic architecture in particular instances does not appear to correlate with factors such as genetic, geographic, or even phenotypic differences among populations [22]–[26]. We know very little about the genetic architecture of quantitative traits. This limits theoretical and practical advances in evolutionary genetics and plant breeding.

Line cross analysis (LCA) is a well established method of quantifying genetic architecture with a long history of use in agriculture. Because of its utility for gene discovery, much recent work has focused on understanding genetic architecture at the level of individual loci or QTL. LCA in contrast measures the summed, i.e. *directional*, effects of all loci contributing to a trait. Line crosses have become more popular in recent years as interest in quantifying epistasis in quantitative traits has increased; this method offers far greater statistical power than variance component analyses previously used to measure epistasis [27]. Traditionally the nearly exclusive realm of plant and animal breeders, LCA have also been used recently to address more broadly evolutionary issues with genetic architecture [28], and are likely to continue to become more common in evolutionary research for several reasons. Demuth and Wade [29], refined by [30], have shown how line crosses between populations can be used to study speciation and Haldane's rule. Directional dominance effects are a requirement for inbreeding depression ([31], p. 257). Hansen and colleagues [2], [32], [33] have shown that the directional epistasis revealed by line cross analysis may be a key to understanding continued response to long term selection, and empirical work in corn and chicken is consistent with this theoretical prediction [34], [35], [12]. The selection responses in corn oil concentration and chicken body weight are also consistent with a large number of loci each with several alleles of small effect [36], [37] and with a large input of new variation from mutation [38]. Clearly we need empirical measures of both locus-specific as well as directional genetic architecture estimates (particularly positive directional epistasis, [2]) to determine the relative roles of these hypothesized factors.

In this paper we present a novel approach to quantifying genetic architecture that combines recombinant inbred lines (RIL) with line cross analysis. When RIL are used in line cross analyses, scaling tests can be constructed for non-additive genetic effects with far more precision than traditional methods of estimation. The RIL can be simultaneously used for QTL identification. These two uses of a RIL population yield qualitatively different information about genetic architecture and can be used in a powerful and complementary manner.

## Materials and Methods

Line cross analyses typically begin with two inbred parental lines that are crossed to produce an F1 generation. F2s, backcrosses, and other generations can be produced as well; the directional genetic effects (also called ‘composite genetic effects’) to be estimated are limited by the number of generation means measured. For example, estimating the mean, additive, dominance, and 3 pairwise epistatic effects requires at least 6 generation means for estimation and 7 for hypothesis testing.

Line cross analyses are primarily carried out using frameworks based on the F2 model of Cockerham [39] or on the F-infinity model of Hayman and Mather [40]. Here we follow line cross theory based on the F2 model as described by Lynch [41] and Lynch and Walsh [31]. We refer to it as the F2 model for simplicity. In this model, the F2 is the reference generation relative to which all genetic effects are derived by linear contrasts. Line crosses use linear combinations of generation phenotypic means to estimate composite genetic effects and carry out significance tests. Each generation mean can be written as a function of two coefficients, the source index (θ_S_) and the hybridity index (θ_H_), multiplied by the additive (A), dominance (D) or epistatic interaction effects (AA, AD, DD, etc.) that potentially differentiate the parental lines (equation 1).
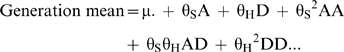
(1)μ. = the mean of the F2 generation. The source index θ_S_ determines the coefficients of the additive effects' contribution to each generation's phenotypic mean. The source index is scaled from one to negative one and indicates the proportion of genes in the generation that came from parent one (P1), with +1 indicating 100% and −1 indicating 0%. P1's θ_S_ = +1 while for F1s, F2s, and RILs θ_S_ = 0.

**Figure 1:**
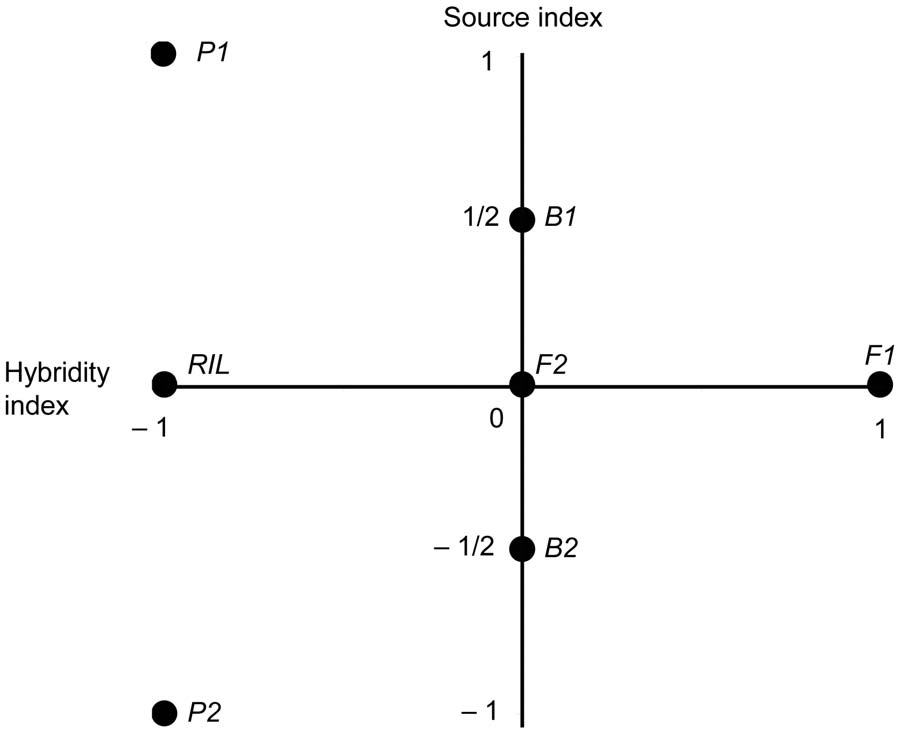
Source and hybridity indices for the various generations of a line cross population. The vertical axis indicates each generation's source index. A source index value of +1 indicates that all genes originate with P1 while −1 indicates that all genes originate with P2. The horizontal axis indicates a generation's hybridity index such that +1 indicates heterozygosity at every locus, while −1 indicates homozygosity at all loci. The RIL values represent an ideal in which an infinite number of generations of selfing preceded measurement of the RIL population. Real RIL populations asymptotically approach this value as the number of generations of inbreeding increases.

The hybridity index determines the contribution of the dominance effects to each generation mean. The hybridity index is also scaled from +1 to −1, with +1 indicating that every locus is heterozygous and −1 indicating that every locus is homozygous. F1s thus have θ_H_ = +1, while parents have θ_H_ = −1. Figure 1 shows the source and hybridity indices for the P1, P2, F1, F2, B1 (back-cross to P1), B2 (back-cross to P2).

To this traditional set of line cross generation means, the mean of a RIL generation can be added. In this context, ‘RILs’ or a ‘RIL population’ is a set of genotypes of highly inbred F2 lines. If these genotypes were replicated, the means of each genotype can be used as individuals for calculating the overall RIL generation mean. RILs asymptotically approach complete homozygosity for all loci as the number of generations of inbreeding approaches infinity. In practice, the convention is to use six to eight generations of inbreeding, resulting in ∼99.84 to 99.96% homozygosity respectively. A major advantage of RILs is that the descendents of any one RIL are genetically identical, hence “immortal” (ignoring mutation accumulation), allowing RILs to be marker-genotyped once and phenotyped repeatedly in multiple labs and experiments. In the framework of LCA, RIL can be used to greatly improve power in estimating non-additive genetic effects.

The F2 generation has a value of zero on both the source and hybridity indices. All genetic effects are scaled relative to this F2 generation mean, thus the linear contrasts used to estimate the genetic effects are sometimes called F2 scaling tests. The expected mean of the F2 and RIL generations are identical and their source indices are both zero (the actual source index for RIL can be approximated from marker data as 2*(number of P1 marker alleles among all lines/total number of alleles)-1, assuming equal spacing of markers throughout the genome. In the absence of segregation distortion, this will be very close to zero). However the F2 hybridity index has zero value, while the RIL hybridity index is in contrast approximately negative one. (Figure 1 and Table 1).

**Table 1:** Source and hybridity indicies and coefficients of directional genetic effects.

Line	S	H	θ_S_	θ_H_	μ	A	D	AA	AD	DD
P1	1	0	1	−1	1	1	−1	1	−1	1
P2	0	0	−1	−1	1	−1	−1	1	1	1
F1	1/2	1	0	1	1	0	1	0	0	1
F2	1/2	1/2	0	0	1	0	0	0	0	0
B1	3/4	1/2	1/2	0	1	1/2	0	1/4	0	0
B2	1/4	1/2	−1/2	0	1	−1/2	0	1/4	0	0
RIL	1/2	0	0	−1	1	0	−1	0	0	1

Source and hybridity indices and the resulting coefficients for the genetic effects in line cross equations, including all two-way epistatic interactions, after (Lynch and Walsh 1998, Chapter 9). Lines are created by crossing inbred parent 1 (P1) with inbred parent 2 (P2) to produce the F1 and F2 generations as well as reciprocal backcrosses to P1 (B1) and P2 (B2). Recombinant inbred lines (RIL) are formed by repeatedly selfing the F2s. The meaning of the columns: S = proportion of genome from P1; H = proportion of heterozygous loci; θ_S_ = source index, indicating the relative contributions of P1 and P2 to the generation genome; θ_H_ = hybridity index, indicating expected heterozygosity of the generation's genome on a scale of 1 to −1. μ = the mean phenotype of the F2 generation. The values in the remaining columns indicate expected contribution of the column's genetic effect to the phenotype of the row's generation. The effect types: A = additive; D = dominance; AA = dominance by dominance interaction; AD = additive by dominance interaction; DD = dominance by dominance interaction.

Products of the source and hybridity indices determine the coefficients for interactions between additive and dominance effects (i.e. epistasis). For example, the product of the additive coefficient (source index) and the dominance coefficient (hybridity index) is the coefficient for the additive x dominance epistatic effect. The coefficients for additive, dominance and pairwise epistatic effects for 7 commonly used generation means are given in Table 1. The RIL generation mean can used in estimating non-additive effects, since in contrast to the F2 it has a non-zero hybridity coefficient.

Using equation (1) and the first six generations in Table 1, Lynch and Walsh ([31]: Table 9.3, p. 214) produced equations to estimate the following composite genetic effects:
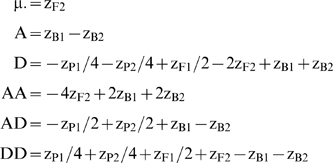
(2)
*z_Xi_* indicates the phenotypic mean of the *X_i_* ^th^ generation (eg. X = B, i = 1 for the B1 generation):

Note the equations for D and AD in Lynch & Walsh ([31] Table 9.3) estimate −D and −AD so we have included the corrected equations here.

By incorporating the RIL generation's equation (for the contributions of the various genetic effects to the RIL generation mean) in the F2 scaling tests, we can construct tests for non-additive genetic effects with fewer terms than traditional tests, shown by contrasting equations (2) and (3). Incorporating the RIL means equalizes the number of generation means necessary to estimate the additive and dominance effects, and the number of generation means necessary to estimate the AA, and DD epistatic effects. This is important in providing equanimity in the power of tests for both intra- and interlocus additive vs. dominance effects; estimates of A and D both require two generation means while AA and DD both require three generation means. AD is the sole equation which retains four generation means in its estimator because the RIL mean cannot be used to simplify the equation.
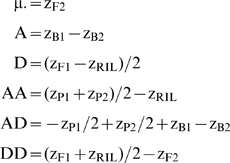
(3)


T-tests can be used to test the null hypothesis that a genetic effect equals zero, assuming that the test statistic is normally distributed under the null hypothesis. The test statistic is simply the estimated genetic effect estimate divided by the standard error of the estimate. For example, the test statistic for the composite dominance effect (using eq. (2)) is
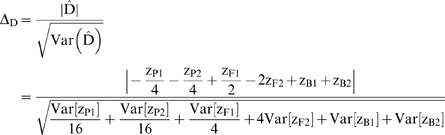
(4)Δ_D_ follows a t distribution with 1 df. Similar test statistics can be constructed for each genetic effect following the same format.

The effect of reducing the number of terms becomes clear when we look at the new RIL-based test statistic for the composite dominance effect:
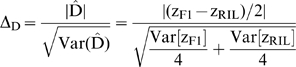
(5)


Recall that the variance of a sum equals the sum of the variances multiplied by the square of the coefficients, i.e. Var (cA + dB) = c^2^ * Var (A) + d^2^ * Var (B), provided that the terms being summed are independent. We can compare the variances associated with the traditional formulae for D, AA, and DD from equations (2) with the corresponding RIL equations (3). For these comparisons, we assume that all generation means have equal variance (i.e. σ^2^ = Var (P1) = Var (P2) = Var (F1) = Var (F2) = Var (B1) = Var (B2) = Var (RIL)).

Based on this assumption, the RIL-based equation for D, AA, and DD have 92%, 94%, and 56% reductions in variance respectively relative to the traditional equations (Table 2). The variance reductions occur for two reasons. First, when fewer generation means are summed to estimate a genetic effect, fewer sources of error are summed into this estimate as well. Second, the RIL equations have smaller coefficients for each generation mean than traditional equations. Since these coefficients are squared when summing the variances, lower coefficients can drastically reduce the variances of the genetic effects. Further variance reductions can occur in RIL based estimates due to the sample size of RIL. Since the number of lines composing the RIL generation is typically large since this determines the power of QTL mapping with RIL populations, the genetic effect variance reduction from using RIL equations is even greater than the reductions using equal variances for all generations illustrated in Table 2. These reductions in variance produce a substantial increase in power to detect dominance and epistasis and to compare dominance-influenced vs. additive effects.

**Table 2:** Comparison of variances of directional genetic effects with and without recombinant inbred lines.

Effect	RIL equation	Variance of RIL-based estimate	Traditional equation	Variance of Traditional estimate	Variance reduction
D	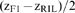	0.5(σ^2^)	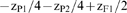 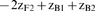	6.375(σ^2^)	92%
AA	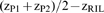	1.5(σ^2^)	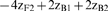	24(σ^2^)	94%
DD	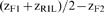	1.5(σ^2^)	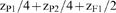 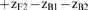	3.375(σ^2^)	56%

RIL-based traditional line cross equations and variance reduction under the assumption of equal variances in the estimate of the means in all generations. Typically, RIL populations will have a lower variance for the estimate of the mean because of their larger sample size. *z*_Xi_ = the phenotypic mean of the Xith generation (eg. *z*_F1_ = mean of the F1 generation).

Frequently, line cross experiments are analyzed using joint scaling tests (e.g. [42]–[44]). The joint scaling test is a weighted least squares regression technique for estimation and significance testing of various models of genetic architecture. A description of this method can be found in [31] (p.215–219, see also: [29], [45]). Briefly, one starts with a vector of generation means (**Y**), a design matrix (**X**) of coefficients derived from the source and hybridity indices, and a vector of composite genetic effects (**β**) to be estimated. Initially, **β** contains the mean and the composite additive effect and **X** contains two corresponding columns. An estimate of **β** is calculated using (**X** ^T^ **X**)^−1^ **X** ^T^ **y** (or (**X^T^V^−1^X**)^−**1**^ **X^T^V^−1^y**, where **V^−1^** is a diagonal matrix of squared standard errors for generation means if sample sizes are unequal). This estimate of **β** is premultiplied by **X** to produce a vector of predicted generation means **Ŷ**, given an additive genetic architecture. **Ŷ** is then compared with the observed **Y** using a chi-squared test. If the observed and predicted **Y**'s are significantly different, then the additive model is rejected and an additive and dominance model is tested next. A new **β** vector containing the mean, the composite additive effect, and the composite dominance effect is estimated and multiplied by an **X** matrix with 3 columns to produce a new **Ŷ**. Increasingly complex models of genetic architecture are tested until the predicted and observed vector of generation means is not significantly different.

To illustrate the advantages of using RIL in a joint scaling context, we used seven generation means to estimate a model of additive, dominance and pairwise epistatic effects:

**Y** = **X** **β**, where
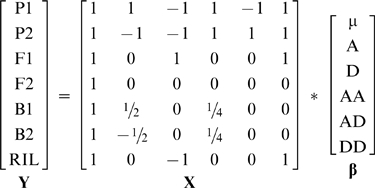
(6)


The general formula for solving linear equations is **β** = (**X** ^T^ **X**)^−1^ **X** ^T^ **y**. When we used Mathematica [46] to solve for **β** in terms of the generation means, the solution is:
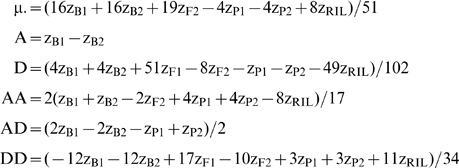
(7)


As in the individual scaling tests, the variance of the dominance effect and the additive x additive effect in RIL models are reduced by 92% and 94% respectively relative to the traditional equations. When the genetic effects are estimated simultaneously using RILs in the model above, the variance of DD is now reduced by 79% (c.f. 56% in individual scaling tests) and the variance of the estimate of the mean is reduced by 63%.

More precise estimation of non-additive genetic effects will help distinguish whether these non-additive effects are rarely detected within micro-evolutionary studies because they are uncommon or because experimental designs have lacked sufficient statistical power to detect them.

## Results and Discussion

Phylogenetically broad crosses have gained increasing importance in both plant breeding and evolutionary genetic studies (e.g. [47]–[49]). Directional gene interactions appear to be increasingly important as the genetic distance between lineages increases. However, even when genetic distances between crossed lines are small, the extent of epistatic interaction can be surprisingly large [25], [42]. Line cross analysis is consequently receiving increased attention as a method for detecting directional gene interactive effects.

We show in this paper that the inclusion of a RIL generation in line cross analysis can greatly increase the accuracy with which D, AA, and DD interactions are estimated. The accurate estimation of gene interaction effects can be of substantial value for those interested in describing genetic architecture and its role in a variety of evolutionary processes [50].

A reviewer has pointed out that one research group has previously incorporated RIL into line cross analysis. Kusterer et al. [51] crossed *Arabidopsis thaliana* C24 and Col-0 genotypes to produce F7 recombinant inbred lines, then crossed these RIL to both parents and F1 in what is known as a triple test cross (TTC) design. RIL, RIL X C24, RIL X Col-0, and RIL X F1 generations were all used in line cross analysis and their results suggested that pairwise and higher order epistasis are important components of the genetic architecture of heterosis for biomass in C24 X Col-0 *Arabidopsis* lines. While the TTC design allows one to estimate non-additive genetic variance components, these additional crosses are not necessary to reap benefits of using RIL in LCA. We suggest purchasing RIL from stock centers to reduce the time consuming crosses necessary for more complex breeding designs.

The reductions in variance used as an illustration in this paper are predicated on the assumption of equal variances in the estimate of every generation line mean. This is not necessarily a realistic assumption, particularly for the RIL generation. First, RIL populations are perforce large. The best RIL populations in many species contain 200–400 RILs and these are often grown and measured in multiple replicates for the purpose of QTL analysis. Line cross generation means are typically calculated with far fewer measures and hence degrees of freedom. Thus we might expect the variance of the mean to be substantially smaller for the RIL mean than for other generations. However, RIL populations very often show transgressive segregation, even when the parents are phenotypically similar. In fact Rieseberg et al. [49] report that 155 of 171 segregating hybrid populations they examined manifested transgressive segregation. We should therefore expect that the F2 and the RIL generations might show higher phenotypic variance than for example the P1, P2, or F1 generations (all three of which are genetically identical within generations and thus will have low variance relative to other generations), and this effect will be exaggerated in RIL compared to the F2 because all individuals are homozygous at virtually all loci. There are thus two offsetting effects on the variance associated with the phenotypic mean of the RIL generation: large sample size reducing the variance of the mean and transgressive segregation and homozygosity increasing the mean's variance. The net effect can only be determined empirically.

If a RIL population is used within a line cross analysis, little extra work is required for QTL mapping. The QTL mapping results will give qualitatively different information on genetic architecture, information that compliments the results of the line cross analysis. QTL mapping can potentially find the number of regions with additive effects (QTL) and the magnitude of those effects, as well as additive x additive epistatic regions responsible for the composite effects detected in line cross analysis. Additionally, QTL mapping may detect loci with equal and opposite effect that are invisible to LCA. For example, if the P1 allele at locus A adds 5 units to the phenotype but the P1 allele at locus B reduces the phenotype by 5 units, LCA will not detect this zero net additive difference between parents. Such canceling effects are clearly often present, evidenced by RIL population parents having very similar phenotypes but widely transgressive segregation in the inbred F2 descendents (reviewed in [52]).

Comparison of additive and additive x additive effects in LCA and QTL analysis can be used to cross validate each result. One would expect that QTL effects summed across the genome will produce a total equal to the composite directional effect produced in LCA. In practice, this may not be the case. QTL analyses are widely known to produce biased results, with QTL number being underestimated and magnitude being over estimated, especially when the number of RIL is small [53], [54]. Differences between composite A and AA effects from LCA and from the summed effects of all QTL discovered may indicate that such biases are present. Additionally, when QTL effects are directional but are too small to be detected by QTL analysis, their sum may still be detected as a difference between means in the line cross analysis. Finally, line cross analysis complements QTL mapping by detecting genetic architecture invisible to QTL analysis. LCA can detect dominance effects and epistatic effects containing dominance that cannot be detected using RIL based QTL mapping.

In summary line cross analysis is a powerful method based on linear contrasts of generation means. Using recombinant inbred lines as a generation in LCA greatly increases the power to detect non-additive genetic effects. Line crosses can detect additive, dominant and epistatic genetic effects of any kind as long as the number of generation means matches or exceeds the number of genetic effects to be estimated. Line cross analysis may detect small genetic effects missed by QTL mapping when effects are directional. QTL mapping using recombinant inbred lines has the ability to detect effects (QTL) of opposite sign invisible to line cross analysis. It can also detect additive and additive by additive epistatic QTL. It can be used to find the location of QTL for effects detected in line cross analysis. Recombinant inbred lines can be purchased from stock centers so that the time and work required to produce them is avoided. QTL studies that wish to incorporate additional line crosses will only require small increase in sample size on the order of 20%. On the other hand, line cross studies will require adding a much larger sample size to add a set of RIL lines large enough for QTL mapping. But these additional organisms phenotyped will not require the time-consuming crosses. Adding line crosses to a QTL experiment or a RIL population to a line cross experiment results in a large increase in ability to measure genetic architecture that will more than justify the modest increase in research effort and cost. Increased statistical power, qualitatively different measures, cross-validation of results, and potential to overcome weaknesses of each approach alone makes this a very powerful approach to gaining a fuller understanding of genetic architecture.

## References

[1] Zheng Z-B, Kao C-H, Basten CJ (1999). Estimating the genetic architecture of quantitative traits.. Genet Res Cambr.

[2] Hansen TF (2006). The evolution of genetic architecture.. Ann Rev Ecol Evol Syst.

[3] Moore JH (2003). The ubiquitous nature of epistasis in determining susceptibility to common human diseases.. Human Heredity.

[4] Cox NJ, Frigge M, Nicolae DL, Concannon P, Hanis CL (1999). Loci on chromosomes 2 (NIDDM1) and 15 interact to increase susceptibility to diabetes in Mexican Americans.. Nature Genetics.

[5] Wiltshire S, Bell JT, Groves CJ, Dina C, Hattersley AT (2006). Epistasis Between Type 2 Diabetes Susceptibility Locion Chromosomes1q21–25 and10q23–26 in Northern Europeans.. Annals of Human Genetics.

[6] Combarros O, Cortina-Borja M, Smith AD, Lehmann DJ (2009). Epistasis in sporadic Alzheimer's disease.. Neurobiology of Aging.

[7] Ankra-Badu GA, Pomp D, Shriner D, Allison DB, Yi N (2009). Genetic influences on growth and body composition in mice: multilocus interactions.. International Journal of Obesity.

[8] Lim U, Peng K, Shane B, Stover PJ, Litonjua AA (2005). Polymorphisms in cytoplasmic serine hydroxymethyltransferase and methylenetetrahydrofolate reductase affect the risk of cardiovascular disease in men.. Journal of Nutrition.

[9] Qin SY, Zhao X, Pan YX, Liu JH, Feng GY (2005). An association study of the N-methyl-D-aspartate receptor NR1 subunit gene (GRIN1) and NR2B subunit gene (GRIN2B) in schizophrenia with universal DNA microarray.. European Journal of Human Genetics.

[10] Wade MJ, Wolf JB, Brodie ED, Wade MJ (2000). Epistasis as a genetic constraint within populations and an accelerant of adaptive divergence among them.. Epistasis and the evolutionary process.

[11] Hallander J, Waldmann P (2007). The effect of non-additive genetic interactions on selection in multi-locus genetic models.. Heredity.

[12] Carlborg O, Jacobsson L, Ahgren P, Siegel P, Andersson L (2006). Epistasis and the release of genetic variation during long-term selection.. Nat Genet.

[13] Weinreich DM, Delaney NF, DePristo MA, Hartl DL (2006). Darwinian evolution can follow only very few mutational paths to fitter proteins.. Science.

[14] Lande R (1980). Sexual dimorphism, sexual selection, and adaptation in polygenic characters.. Evol.

[15] Lande R, Arnold SJ (1983). The measurement of selection on correlated characters.. Evol Evol.

[16] Dobzhansky T (1937). Genetics and the origin of species.

[17] Porter AH, Johnson NA (2002). Speciation despite gene flow when developmental pathways evolve.. Evol.

[18] Gavrilets S (1997). Evolution and speciation on holey adaptive landscapes.. Trends Ecol Evol.

[19] Coyne JA, Orr HA (1997). “Patterns of speciation in Drosophila” revisited.. Evol.

[20] Presgraves DC (2002). Patterns of post-zygotic isolation in Lepidoptera.. Evol.

[21] Kondrashov AS, Sunyaev S, Kondrashov FA (2002). Dobzhansky-Muller incompatibilities in protein evolution.. Proc Nat Acad Sci US A.

[22] Erickson D, Fenster CB (2006). Intraspecific hybridization and the recovery of fitness in the native legume Chamaecrista fasciculata.. Evol.

[23] Fenster CB, Galloway LF (2000). Population differentiation in an annual legume: genetic architecture.. Evol.

[24] Edmands S (2002). Does parental divergence predict reproductive compatibility?. Trends Ecol Evol.

[25] Demuth JP, Wade MJ (2007). Population differentiation in the beetle Tribolium castaneum. I. Genetic architecture.. Evol.

[26] Demuth JP, Wade MJ (2007). Population differentiation in the beetle Tribolium castaneum. II. Haldane's rule and incipient speciation.. Evol.

[27] Demuth JP, Wade MJ (2006). Experimental Methods for Measuring Gene Interactions.. Ann Rev Ecol Evol Syst.

[28] Roff DA, Emerson K, Goodnight C (2006). Epistasis and dominance: evidence for differential effects in life-history versus morphological traits.. Evol.

[29] Demuth JP, Wade MJ (2005). On the theoretical and empirical framework for studying genetic interactions within and among species.. Amer Nat.

[30] Fitspatrick (2008). Fitzpatrick, BÂ M. 2008. Hybrid Dysfunction: Population Genetic and Quantitative Genetic Perspectives.. The American Naturalist.

[31] Lynch M, Walsh B (1998). Genetics and the analysis of quantitative traits.

[32] Carter AJR, Hermisson J, Hansen TF (2005). The role of epistatic gene interactions in the response to selection and the evolution of evolvability.. Theoret Pop Biol.

[33] Wagner GP, Altenberg L (1996). Complex adaptations and the evolution of evolvability.. Evol.

[34] Dudley JW (2007). From Means to QTL: The Illinois Long-Term Selection Experiment as a Case Study in Quantitative Genetics.. Crop Sci.

[35] Carlborg O, Haley CS (2004). Epistasis: too often neglected in complex trait studies?. Nat.

[36] Barton, Keightly (2002). Understanding quantitative genetic variation.. Nat Rev Genet.

[37] Dudley JW (2008). Epistatic Interactions in Crosses of Illinois High Oil x Illinois Low Oil and of Illinois High Protein x Illinois Low Protein Corn Strain.. Crop Sci.

[38] Keightley PD (2004). Mutational Variation and Long-term Selection Response.. Plant Breeding Reviews.

[39] Cockerham CC (1954). An extension of the concept of partitioning hereditary variance for analysis of covariances among relatives when epistasis is present.. Genet.

[40] Hayman BI, Mather K (1955). The description of genetic interactions in continuous variation.. Biomet.

[41] Lynch M (1991). The genetic interpretation of inbreeding depression and outibreeding depresssion.. Evolution.

[42] Kelly JK (2005). Epistasis in monkeyflowers.. Genet.

[43] Sun Z, Lower RL, Staub JE (2006). Analysis of generation means and components of variance for parthenocarpy in cucumber (Cucumis sativus L.).. Plant Breeding.

[44] Wegner KM, Berenos C, Schmid-Hempel P (2008). Nonadditive genetic components in resistance of the red flour beetle Tribolium castanaeum against parasite infection.. Evol.

[45] Bradshaw WE, Holzapfel CM, Wolf JB, Brodie ED, Wade MJ (2000). The evolution of genetic architectures and the divergence of natural populations.. Epistasis and the evolutionary process.

[46] Champaign IL, Wolfram Research, Inc., Mathematica, Version 6.0 (2007).

[47] Schemske DW, Bradshaw HD (1999). Pollinator preference and the evolution of floral traits in monkeyflowers (Mimulus).. Proc Nat Acad Sci USA.

[48] López-Fernández H, Bolnick DI (2007). What causes partial F1 Hybrid viability? Incomplete penetrance versus genetic variation.. PLoS ONE.

[49] Rieseberg LH, Archer MA, Wayne RK (1999). Transgressive segregation, adaptation and speciation.. Hered.

[50] Whitlock MC, Phillips PC, Moore FB-G, Tonsor SJ (1995). Multiple fitness peaks and epistasis.. Ann Rev Ecol Evol Syst.

[51] Kusterer B, Muminovic J, Utz HF, Piepho HP, Barth S, Heckenberger M, Meyer RC, Altmann T, Melchinger AE (2007). Analysis of a triple testcross design with recombinant inbred lines reveals a significant role of epistasis in heterosis for biomass-related traits in Arabidopsis.. Genetics.

[52] Tonsor SJ, Alonso-Blanco C, Koornneef M (2005). Gene function beyond single traits: the evolutionary ecology of genetic effects in Arabidopsis thaliana.. Plant Cell Envir.

[53] Beavis WD, Patterson AH (1998). QTL Analyses: Power, Precision, and Accuracy.. Molecular dissection of complex traits.

[54] Xu S (2003). Theoretical Basis of the Beavis Effect.. Genet.

